# Characteristics of Computed Tomography for Identifying Patients at High Risk of Endogenous Endophthalmitis Due to *Klebsiella pneumoniae*-Related Pyogenic Liver Abscess

**DOI:** 10.3390/jcm11154376

**Published:** 2022-07-28

**Authors:** Jae Jung Lee, Seung Baek Hong, Nam Kyung Lee, Young Joo Park, So Hee Kim, Sung Who Park, Iksoo Byon, Suk Kim

**Affiliations:** 1 Department of Ophthalmology, Biomedical Research Institute, Pusan National University Hospital, School of Medicine, Pusan National University, Pusan 46241, Korea; otjj85@naver.com (J.J.L.); sohee818@naver.com (S.H.K.); oph97@naver.com (S.W.P.); isbyon@naver.com (I.B.); 2 JRYN Eye Clinic, Pusan 47296, Korea; 3 Department of Radiology, Biomedical Research Institute, Pusan National University Hospital, School of Medicine, Pusan National University, Pusan 46241, Korea; leenk77@hanmail.net (N.K.L.); kimsuk8819@gmail.com (S.K.); 4 Department of Internal Medicine, Biomedical Research Institute, Pusan National University Hospital, School of Medicine, Pusan National University, Pusan 46241, Korea; juya0630@naver.com; 5 Lee Eye Clinic, Pusan 611831, Korea

**Keywords:** computed tomography, diabetes mellitus, endogenous endophthalmitis, liver abscess, *Klebsiella pneumoniae*

## Abstract

Endogenous endophthalmitis (EE) associated with *Klebsiella pneumoniae* (*K. pneumoniae*)-related pyogenic liver abscess (PLA) is one of the fatal complications of PLA and leads to loss of vision. Early diagnosis and treatment are important to save the patient’s vision. We investigated the characteristics of computed tomography (CT) in EE associated with *K. pneumoniae*-related PLA for the identification of the predictors of EE, in order to facilitate early diagnosis. A total of 274 patients diagnosed with *K. pneumoniae*-related PLA, including 15 patients with EE, were identified between January 2005 and December 2019. The clinical (age, gender, and underlying disease) and radiologic (the location, size, and number of abscesses) features were reviewed. In addition, the involvement of the adjacent vessels, such as the hepatic vein and portal vein, was carefully reviewed. A comparative analysis was performed between the EE and non-EE groups. Uni- and multivariate logistic regression analyses were used to identify the predictors of EE. Diabetes mellitus (DM), the involvement of the left or both hepatic lobes, and the adjacent vessels on the CT were significantly more frequent than those in the non-EE group (*p* < 0.05 in all), and they were the significant predictors of EE in the logistic regression analyses. In patients with *K. pneumoniae*-related PLA, the CT findings, such as the locations of the abscess (i.e., left or both lobes) and the involvement of the adjacent vessels, should be considered in addition to the ocular symptoms for an early diagnosis of EE.

## 1. Introduction

Pyogenic liver abscess (PLA) is a rare but potentially life-threatening disease. Organisms from PLA can spread to the bloodstream and metastasize to multiple organs. Endogenous endophthalmitis (EE) is a rare but fatal complication of PLA that can lead to loss of vision.

The distribution of the causative organisms of EE due to PLA differs according to the regional differences between Western and East Asian countries. Gram-positive bacteria, such as *Staphylococcus aureus*, *Streptococci*, and *Candida*, are the main pathogens in Western countries, whereas Gram-negative bacteria, such as *Klebsiella pneumoniae* (*K. pneumoniae*), are the most common pathogens in East Asian countries [1,2,3,4].

Previous studies have reported on the clinical characteristics and risk factors of EE associated with PLA, such as old age, diabetes mellitus (DM), and initial visual acuity (VA). In addition, *K. pneumoniae* infection was also reported to be a risk factor for EE associated with PLA [5,6,7,8,9,10].

Poor visual prognosis has also been reported in patients with EE from *K. pneumoniae*-related PLA. Poor or worse VA of light perception was reported in 89% of the patients’ eyes in the study, and 41% of these were eventually eviscerated or enucleated [7]. Therefore, early diagnosis of EE and prompt treatment are needed to improve the visual prognosis and save the patient’s vision.

Several studies have reported that the location of PLA is associated with EE [6,11]. Imaging tools, such as computed tomography (CT) and ultrasonography, are used to determine the location of PLA. Identifying the risk of EE before the onset of symptoms when using imaging tools for the diagnosis of PLA would be beneficial. However, specific CT findings that could be utilized to identify a high risk of EE in patients with *K. pneumoniae*-related PLA have not been reported.

Thus, this study aimed to investigate the CT characteristics of patients with EE associated with *K. pneumoniae*-related PLA and to find the predictors of EE.

## 2. Materials and Methods

The retrospective study was approved by the Institutional Review Board of our institute (IRB No. 2002-009-088), and informed consent was waived due to the retrospective nature of this study.

### 2.1. Study Patients

**Figure 1:**
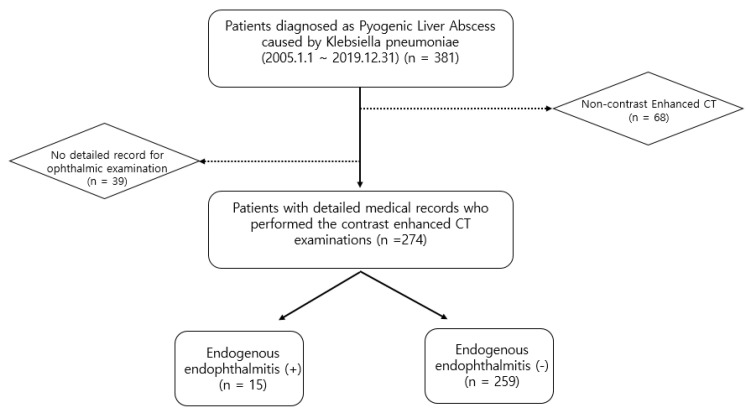
Flow diagram of the inclusion and exclusion criteria.

Using the electronic database of our institute, 381 patients diagnosed with *K. pneumoniae*-related PLA between 1 January 2005 and 31 December 2019 were identified. Among these 381 patients, we excluded 107 patients for the following reasons: (a) incomplete medical records for ophthalmic examinations, such as the slit-lamp and fundus examinations, and (b) non-contrast-enhanced CT examination. The study included (a) patients who underwent contrast-enhanced CT examination, and (b) patients with detailed medical records who underwent ophthalmic examination for suspected EE. Thus, 274 patients were included in this study. Fifteen patients (17 eyes) were diagnosed with EE (Figure 1). Patients were diagnosed with EE if they met the following criteria: (1) the presence of anterior chamber inflammation, vitritis, a subretinal abscess, or a choroidal abscess; (2) a culture positive for blood, vitreous or aqueous; and (3) no other possible causes of endophthalmitis, such as previous ophthalmic surgery, a corneal ulcer, or ocular trauma.

### 2.2. Medical Record Review

An expert ophthalmologist (J.J.L.; board-certified retinal specialist; 10 years of clinical experience) reviewed the demographic and clinical data, including age, gender, underlying diseases, and presence of endophthalmitis, for the included patients.

### 2.3. Imaging Analysis

Contrast-enhanced CT examinations of the included patients were reviewed by an expert radiologist (S.B.H.; board-certified radiologist; 10 years of clinical experience) to determine the location (segment of the lobe), size, and number (single or multiple) of the abscesses.

The involvement of the adjacent vessels, such as the hepatic and portal veins, was also carefully reviewed by two expert radiologists (S.B.H. and N.K.L.; board-certified radiologists; 10 and 19 years of clinical experience, respectively). The discordant cases for the presence of involvement of adjacent vessels were resolved by consensus.

### 2.4. Statistical Analysis

A comparative analysis was performed between the EE and non-EE groups. The clinical and radiologic features were compared between the two groups. After conducting Shapiro–Wilk test and Kolmogorov–Smirnov tests, which are normality tests, the Student’s t-test was used to compare the continuous variables between the two groups. To compare the categorical variables, Fisher’s exact test or the Chi-square test were used.

Logistic regression analysis was used to identify the predictors of EE. *p*-values < 0.05 were considered statistically significant.

Inter-reader agreement for the presence of involvement of the adjacent vessels was assessed using kappa statistics.

All statistical analyses were performed using IBM SPSS Statistics for Windows, Version 23.0 (IBM Corp., Armonk, NY, USA).

## 3. Results

### 3.1. Patient Characteristics

A total of 274 patients were included in this study. The mean age was 69.1 ± 13.03 years. Among the 274 patients, 174 patients were male and 100 patients were female. A single abscess was observed in 232 patients and multiple abscesses were observed in 42 patients. The average size was 5.87 ± 2.44 cm. The abscess was located in the right lobe in 182 patients, the left lobe in 71 patients, and both lobes in 21 patients. The cT findings in 50 patients showed the involvement of the adjacent vessels. DM was observed in 28.1%. These demographics and CT findings are summarized in Table 1.

EE was identified in 15 patients (17 eyes) among the 274 patients. The CT examinations of all patients with EE associated with *K. pneumoniae*-related PLA satisfied at least one of the following criteria: (1) the size of the abscess was over 5 cm, and (2) there was involvement of the adjacent hepatic or portal veins.

### 3.2. Comparison between the EE and Non-EE Groups

The mean age was 67.47 ± 11.51 years in the EE group and 69.19 ± 13.12 years in the non-EE group. Ten patients (66.7%) were male and five were female in the EE group; 164 patients (63.3%) were male and 95 patients were female in the non-EE group. A single abscess was observed in 12 of the 15 patients in the EE group (66.7%) and 220 of the 259 patients in the non-EE group (84.9%). The average size of the abscess was 6.0 ± 2.30 cm in the EE group and 5.9 ± 2.45 cm in the non-EE group. The location of the abscess differed significantly between the EE and non-EE groups (*p* = 0.005). An abscess located in the left lobe was more frequent in the EE group (53.3%), whereas an abscess located in the right lobe was more frequent in the non-EE group (68.7%). The involvement of the adjacent vessels was significantly more frequent in the EE group (*p* < 0.001). DM was significantly more common in the EE group (*p* = 0.002). The comparison of the demographics and CT findings between the EE and non-EE groups is shown in Table 2.

### 3.3. Clinico-Radiologic Predictors for EE

**Figure 2:**
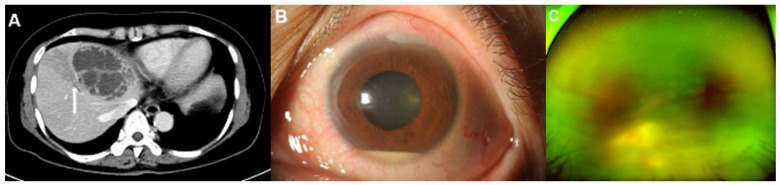
Representative images of a 59-year-old woman with diabetes mellitus who presented with endogenous endophthalmitis due to *Klebsiella pneumoniae*-related pyogenic liver abscess. (**A**) A contrast-enhanced computed tomography (CT) image showed a 7.5 cm septated cystic lesion, representing a *Klebsiella* hepatic abscess, at liver segment IV. The CT image also showed thrombophlebitis on the adjacent hepatic vein due to direct invasion from the hepatic abscess (arrow). (**B**) Hyperemia and a 2 mm hypopyon in the anterior chamber were observed in the slit-lamp examination. (**C**) A whitish mass-like lesion, a suspected subretinal abscess, was noticed on wide fundus photograph. Both the slit-lamp examination and the fundus photograph indicated signs of endophthalmitis.

**Figure 3:**
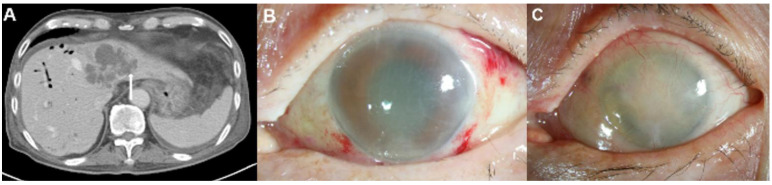
Representative images of an 87-year-old man with diabetes mellitus who presented endogenous endophthalmitis due to *Klebsiella pneumoniae*-related pyogenic liver abscess. (**A**) This patient had biliary cirrhosis due to recurrent pyogenic cholangitis and underwent contrast computed tomography (CT) examination. The CT image showed a 6.8 cm septated cystic lesion, representing a *Klebsiella* hepatic abscess, at liver segments II and IV. The CT image also showed the hepatic abscess with invasion from the adjacent hepatic vein (arrow). (**B**) Severe corneal edema and anterior chamber haziness were noted during an initial slit-lamp examination without history of previous ocular surgery. (**C**) Despite aggressive treatment, his eye developed phthisis.

Univariate and multivariate logistic regression analyses were performed to identify the predictors of EE. In the univariate logistic regression analysis, left-lobe location (*p* = 0.006, odds ratio (OR) = 5.651), location in both lobes (*p* = 0.013, OR = 7.417), the involvement of the adjacent vessels (*p* < 0.001, OR = 39), and DM (*p* = 0.002, OR = 5.731) were identified as predictors of EE. Multiplicity and a size > 5 cm were not associated with EE (*p* = 0.607 and *p* = 0.662, respectively). In the multivariate logistic regression analysis, left-lobe location (*p* = 0.002, OR = 19.622), location in both lobe (*p* = 0.002, OR = 45.107), the involvement of the adjacent vessels (*p* < 0.001, OR = 148.107), and DM (*p* = 0.004, OR = 11.628) were identified as significant predictors of EE (Figure 2 and Figure 3) (Table 3).

### 3.4. Inter-Reader Agreement for Assessing the Presence of Involvement of the Adjacent Vessels

The inter-reader agreement for assessing the presence of involvement of the adjacent vessels was very good. The kappa value was 0.872 (*p* < 0.001).

## 4. Discussion

EE is an extremely rare but serious complication of PLA. The incidence of EE has been reported to vary from 0.005% to 1.92% [1,6,11,12,13,14,15]. Due to its rare prevalence, there have not been many studies with high confidence on the risk factors for EE associated with PLA. However, EE is a vision-threatening emergency, necessitating early diagnosis. Our study showed that left-lobe location (*p* = 0.002, OR = 19.622), location in both lobes (*p* = 0.002, OR = 45.107), the involvement of the adjacent vessels (*p* < 0.001, OR = 148.107), and DM (*p* = 0.004, OR = 11.628) were significant predictors of EE in patients with *K. pneumoniae*-related PLA.

Previous studies have identified DM, old age, *Klebsiella* species infection, and immune insufficiency as known risk factors for EE [5,6,7,8,9,10]. Several studies involving the Korean population reported DM to be the most common risk factor [16,17,18,19]. Similarly to these studies, DM was the most common (66.7%) underlying disease in our study, and it was also identified as a risk factor. Hyperglycemia associated with DM suppresses polymorphonuclear functions and increases the risk of vascular complications and infection [20]. Wang et al. also reported that an HbA1c concentration > 9.0% was associated with hepatic venous thrombophlebitis and metastatic infection in patients with *K. pneumoniae*-related PLA [21].

EE associated with *K. pneumoniae*-related PLA is associated with poor visual outcomes [5,7]. Gram-negative *K. pneumoniae* is the predominant causative microorganism in East Asian countries [1,3,4,22,23,24], and its prevalence has been increasing worldwide. The organs most susceptible to *K. pneumoniae* infection, other than the liver, are the lungs, eyes, and central nervous system [25]. Therefore, the incidence of EE associated with PLA has increased significantly in East Asia recently [4,5,26,27,28]. *Klebsiella* forms a mucus-rich biofilm that is resistant to phagocytosis by macrophages and neutrophils and penetrates the blood–ocular barrier. Due to the direct intraocular invasion of this bacteria, the prognosis is poorer than that of other bacterial infections [29,30]. Meyers-Elliot et al. reported that *Klebsiella*-induced endophthalmitis led to irreversible and rapid destruction of the retinal photoreceptors within 48 h of infection in an experimental rabbit model [31].

Yang et al. reported that among the patients with EE associated with *K. pneumoniae*-related PLA, 89% had poor or worse VA of light perception and 41% underwent ophthalmectomies despite aggressive treatment for EE [7]. Thus, EE is a fatal condition that progresses rapidly despite aggressive treatment with appropriate antibiotics or surgery [32]. Immediate treatment within 24 h and early intervention, such as a vitrectomy with intravitreal antibiotic injection, may improve visual outcomes [32,33].

Identifying the predictors of EE before the onset of ocular symptoms in patients diagnosed with *K. pneumoniae*-related PLA would facilitate rapid diagnosis and treatment. CT is a good imaging modality used for the early diagnosis of PLA. Lee et al. reported the significant imaging predictors of *K. pneumoniae*-related PLA. CT findings, such as thin-walled abscesses, metastatic infection, the absence of rim enhancement, and the absence of an underlying biliary tract disease, can be used for the early diagnosis of *K. pneumoniae*-related PLA [34]. However, endophthalmitis was observed in only one patient among the 219 patients in their study, and they could not evaluate the specific CT findings for EE associated with *K. pneumoniae*-related PLA. In our study, the location of the abscess in the left or both lobes of the liver and the involvement of the adjacent vessels were identified as risk factors for EE.

Our results are somewhat different from those of other studies. Rahiman et al. reported that the right lobe of the liver is the most likely site of infection because of the unequal distribution of the superior and inferior mesenteric vein contents [35]. Park et al. also reported that the location of the abscess in the right superior segment was a risk factor for liver-abscess-associated EE [11]. They explained that metastatic infections are propagated via the inferior vena cava (IVC), and the right superior segment is close to the IVC. Meanwhile, Lee et al. reported that the most frequent location of liver abscesses associated with EE was the left lobe, a finding which was similar to the findings of our study [6]. There are several studies with high heterogeneity regarding the location of abscess.

The presence of involvement of the adjacent vessels must be considered. The involvement of the adjacent vessels, such as hepatic-vein or portal-vein infiltration, suggests metastatic infection. EE is considered the most common and serious septic complication of PLA. Therefore, the ocular examination for EE should be suggested if involvement of the adjacent vessels is observed.

This study has several limitations. Firstly, our study had a retrospective design; therefore, selection bias was present. However, we collected all the data consecutively. Secondly, our study included a relatively small number of patients with EE. The incidence of EE associated with *K. pneumoniae*-related PLA was low. However, we identified significantly different factors through comparative analysis between the EE group and the non-EE group, and also significant predictors of EE. Thirdly, DM is a frequent comorbidity of PLA, and this high incidence may have influenced the statistical analysis performed.

## 5. Conclusions

In summary, the early diagnosis of EE in patients with *K. pneumoniae*-related PLA is important for saving the eye. Patients diagnosed with *K. pneumoniae*-related PLA should undergo ophthalmic examination, even in the absence of ocular symptoms. However, if the patient’s condition makes it difficult to perform an ocular examination, CT findings of PLA, such as adjacent vessel involvement, could help to identify EE. Moreover, patients with DM should be carefully evaluated to identify EE and other septic conditions.

## Figures and Tables

**Table 1:** Demographics and computed tomography findings of patients with pyogenic liver abscess caused by *Klebsiella pneumoniae*.

Parameters	Results
Age (years) Gender (male/female)	69.1 ± 13.03
	174/100
Number of abscesses (single/multiple)	232/42
Size of abscesses (cm)	5.87 ± 2.44
Location of abscesses (right/left/both)	182/71/21
Involvement of adjacent hepatic or portal veins (+/−)	50/224
Diabetes mellitus (+/−)	77/197

**Table 2:** Comparison of the demographics and computed tomography findings between the endogenous endophthalmitis group and non-endogenous endophthalmitis group in patients with *Klebsiella pneumoniae* liver abscess.

Parameters	EE Group	Non-EE Group	*p*-Value
Age (years)	67.47 ± 11.51	69.19 ± 13.12	0.619 ^a^
Gender (male/female)	10/5	164/95	1.000 ^b^
Number of abscesses (single/multiple)	12/3	220/39	0.710 ^b^
Size of abscesses (cm)	6.0 ± 2.30	5.9 ± 2.45	0.838 ^a^
Location of abscesses (right/left/both)	4/8/3	178/63/18	0.005 ^c,^*
Involvement of adjacent hepatic or portal veins (+/−)	13/2	37/222	<0.001 ^b,^*
Diabetes mellitus (+/−)	10/5	67/192	0.002 ^b,^*

EE = endogenous endophthalmitis. ^a^ The *p*-value was obtained via the Student’s *t*-test. ^b^ The *p*-value was obtained via the Fisher’s exact test. ^c^ The *p*-value was obtained via the Chi-square test. * indicates statistically significant differences at *p* < 0.05.

**Table 3:** Logistic regression analysis of the risk factors for endogenous endophthalmitis.

		Univariate Analysis		Multivariate Analysis	
		Odds Ratio	*p*-Value	Odds Ratio	*p*-Value
Multiplicity		1.41	0.607		
Location	Right	1 *		1 *	
	Left	5.651	0.006	19.622	0.002
	Both	7.417	0.013	45.107	0.002
Size > 5 cm		1.278	0.662		
Involvement of adjacent hepatic or portal veins		39	<0.001	148.107	<0.001
Diabetes mellitus (+/−)		5.731	0.002	11.628	0.004

* Reference for calculating the odds of the other subcategories of a variable.

## Data Availability

The data that support the findings of this study are available on request to the corresponding author. The data are not publicly available due to privacy issues.

## References

[1] Okada A.A., Johnson R.P., Liles W.C., D’Amico D.J., Baker A.S. (1994). Endogenous bacterial endophthalmitis. Report of a ten-year Retrospective Study. Ophthalmology.

[2] Durand M.L. (2013). Endophthalmitis. Clin. Microbiol. Infect..

[3] Lingappan A., Wykoff C.C., Albini T.A., Miller D., Pathengay A., Davis J.L., Flynn H.W. (2012). Endogenous fungal endophthalmitis: Causative organisms, management strategies, and visual acuity outcomes. Am. J. Ophthalmol..

[4] Wong J.S., Chan T.K., Lee H.M., Chee S.P. (2000). Endogenous bacterial endophthalmitis: An east Asian experience and a reappraisal of a severe ocular affliction. Ophthalmology.

[5] Chen Y.J., Kuo H.K., Wu P.C., Kuo M.L., Tsai H.H., Liu C.C., Chen C.H. (2004). A 10-year comparison of endogenous endophthalmitis outcomes: An east Asian experience with Klebsiella pneumoniae infection. Retina.

[6] Lee J.Y., Kim K.H. (2014). Endogenous endophthalmitis complicated by pyogenic liver abscess: A review of 17 years’ experience at a single center. Digestion.

[7] Yang C.S., Tsai H.Y., Sung C.S., Lin K.H., Lee F.L., Hsu W.M. (2007). Endogenous Klebsiella endophthalmitis associated with pyogenic liver abscess. Ophthalmology.

[8] Relhan N., Forster R.K., Flynn H.W. (2018). Endophthalmitis: Then and Now. Am. J. Ophthalmol..

[9] Jackson T.L., Eykyn S.J., Graham E.M., Stanford M.R. (2003). Endogenous bacterial endophthalmitis: A 17-year prospective series and review of 267 reported cases. Surv. Ophthalmol..

[10] Greenwald M.J., Wohl L.G., Sell C.H. (1986). Metastatic bacterial endophthalmitis: A contemporary reappraisal. Surv. Ophthalmol..

[11] Park I.H., Jun C.H., Wi J.W., Park S.Y., Lee W.S., Jung S.I., Park C.H., Joo Y.E., Kim H.S., Choi S.K. (2015). Prevalence of and risk factors for endogenous endophthalmitis in patients with pyogenic liver abscesses. Korean J. Intern. Med..

[12] Tan Y.M., Chee S.P., Soo K.C., Chow P. (2004). Ocular manifestations and complications of pyogenic liver abscess. World J. Surg..

[13] Hu C.C., Ho J.D., Lou H.Y., Keller J.J., Lin H.C. (2012). A one-year follow-up study on the incidence and risk of endophthalmitis after pyogenic liver abscess. Ophthalmology.

[14] Chung K.S., Kim Y.K., Song Y.G., Kim C.O., Han S.H., Chin B.S., Gu N.S., Jeong S.J., Baek J., Choi B.J. (2011). Clinical review of endogenous endophthalmitis in Korea: A 14-year review of culture positive cases of two large hospitals. Yonsei Med. J..

[15] Lee S.J., Lee M.A., Kwak H.W. (2000). Clinical aspect of bacterial endogenous endophthalmitis. J. Korean Ophthalmol. Soc..

[16] Hwang J.H., Cho N.C. (2009). Prognostic factors in patients with endogenous endophthalmitis. J. Korean Ophthalmol. Soc..

[17] Lee S., Um T., Joe S.G., Hwang J.U., Kim J.G., Yoon Y.H., Lee J.Y. (2012). Changes in the clinical features and prognostic factors of endogenous endophthalmitis: Fifteen years of clinical experience in Korea. Retina.

[18] Lim H.W., Shin J.W., Cho H.Y., Kim H.K., Kang S.W., Song S.J., Yu H.G., Oh J.R., Kim J.S., Moon S.W. (2014). Endogenous endophthalmitis in the Korean population: A six-year retrospective study. Retina.

[19] Cho H., Shin Y.U., Siegel N.H., Yu H.G., Sobrin L., Patel A., Durand M.L., Miller J.W., Husain D. (2018). Endogenous Endophthalmitis in the American and Korean Population: An 8-year Retrospective Study. Ocul. Immunol. Inflamm..

[20] Delamaire M., Maugendre D., Moreno M., Le Goff M.C., Allannic H., Genetet B. (1997). Impaired leucocyte functions in diabetic patients. Diabet. Med..

[21] Wang H.H., Tsai S.H., Yu C.Y., Hsu H.H., Liu C.H., Lin J.C., Huang G.S., Cheng W.T., Tung H.J., Chen C.Y. (2014). The association of haemoglobin A₁C levels with the clinical and CT characteristics of Klebsiella pneumoniae liver abscesses in patients with diabetes mellitus. Eur. Radiol..

[22] Jackson T.L., Paraskevopoulos T., Georgalas I. (2014). Systematic review of 342 cases of endogenous bacterial endophthalmitis. Surv. Ophthalmol..

[23] Odouard C., Ong D., Shah P.R., Gin T., Allen P.J., Downie J., Lim L.L., McCluskey P. (2017). Rising trends of endogenous Klebsiella pneumoniae endophthalmitis in Australia. Clin. Exp. Ophthalmol..

[24] Kashani A.H., Eliott D. (2013). The emergence of Klebsiella pneumoniae endogenous endophthalmitis in the USA: Basic and clinical advances. J. Ophthalmic. Inflamm. Infect..

[25] Siu L.K., Yeh K.M., Lin J.C., Fung C.P., Chang F.Y. (2012). Klebsiella pneumoniae liver abscess: A new invasive syndrome. Lancet Infect. Dis..

[26] Sheu S.J., Kung Y.H., Wu T.T., Chang F.P., Horng Y.H. (2011). Risk factors for endogenous endophthalmitis secondary to klebsiella pneumoniae liver abscess: 20-year experience in Southern Taiwan. Retina.

[27] Tsai F.C., Huang Y.T., Chang L.Y., Wang J.T. (2008). Pyogenic liver abscess as endemic disease, Taiwan. Emerg Infect Dis..

[28] Zhu X., Wang S., Jacob R., Fan Z., Zhang F., Ji G. (2011). A 10-year retrospective analysis of clinical profiles, laboratory characteristics and management of pyogenic liver abscesses in a chinese hospital. Gut. Liver.

[29] Hunt J.J., Wang J.T., Callegan M.C. (2011). Contribution of mucoviscosity-associated gene A (magA) to virulence in experimental Klebsiella pneumoniae endophthalmitis. Investig. Ophthalmol. Vis. Sci..

[30] Wiskur B.J., Hunt J.J., Callegan M.C. (2008). Hypermucoviscosity as a virulence factor in experimental Klebsiella pneumoniae endophthalmitis. Investig. Ophthalmol. Vis. Sci..

[31] Meyers-Elliott R.H., Dethlefs B.A. (1982). Experimental Klebsiella-induced endophthalmitis in the rabbit. Arch. Ophthalmol..

[32] Yoon Y.H., Lee S.U., Sohn J.H., Lee S.E. (2003). Result of early vitrectomy for endogenous Klebsiella pneumoniae endophthalmitis. Retina.

[33] Chou F.F., Kou H.K. (1996). Endogenous endophthalmitis associated with pyogenic hepatic abscess. J. Am. Coll. Surg..

[34] Lee J.H., Jang Y.R., Ahn S.J., Choi S.J., Kim H.S. (2020). A retrospective study of pyogenic liver abscess caused primarily by Klebsiella pneumoniae vs. non-Klebsiella pneumoniae: CT and clinical differentiation. Abdom. Radiol..

[35] Rahimian J., Wilson T., Oram V., Holzman R.S. (2004). Pyogenic liver abscess: Recent trends in etiology and mortality. Clin. Infect. Dis..

